# Self-Enhanced Carbonized Polymer Dots for Selective Visualization of Lysosomes and Real-Time Apoptosis Monitoring

**DOI:** 10.1016/j.isci.2020.100982

**Published:** 2020-03-13

**Authors:** Xiaohuan Zhao, Jing Li, Dongning Liu, Mingxi Yang, Wenjing Wang, Shoujun Zhu, Bai Yang

**Affiliations:** 1 State Key Laboratory of Supramolecular Structure and Materials, College of Chemistry, Jilin University, Changchun, Jilin 130012, P. R. China; 2 The Scientific Research Center, China-Japan Union Hospital, Jilin University, Changchun, Jilin 130033, P. R. China; 3 Department of Periodontology, Stomatology Hospital, Jilin University, Changchun, Jilin 130021, P. R. China; 4 Key Laboratory of Organ Regeneration & Transplantation of the Ministry of Education, The First Hospital of Jilin University, Changchun, Jilin 130061, P.R. China

**Keywords:** Organic Chemistry, Optical Materials, Biotechnology

## Abstract

Protons are highly related to cell viability during physiological and pathological processes. Developing new probes to monitor the pH variation could be extremely helpful to understand the viability of cells and the cell death study. Carbonized polymer dots (CPDs) are superior biocompatible and have been widely applied in bioimaging field. Herein, a new type of extreme-pH suitable CPDs was prepared from citric acid and o-phenylenediamine (CA/oPD-CPDs). Due to the co-existence of hydrophilic and hydrophobic groups, CA/oPD-CPDs tend to aggregate in neutral condition with a dramatic decrease of fluorescence, but disperse well in both acidic and alkaline conditions with brighter emission. This specialty enables them to selectively illuminate lysosomes in cells. Moreover, CA/oPD-CPDs in the cytoplasm could serve as a sustained probe to record intracellular pH variation during apoptosis. Furthermore, CA/oPD-CPDs present a continuous fluorescence increase upon 2-h laser irradiation in living cells, underscoring this imaging system for long-term biological recording.

## Highlights

• CA/oPD-CPDs with extreme pH sensitivity through aggregation-dissociation process
• Irradiation-enhanced CA/oPD-CPDs for mapping living cells in a real-time manner
• CA/oPD-CPDs super-nanoparticles assembly to selectively visualize lysosomes
• Mapping the spatial distribution of pH in living cells during apoptosis

## Graphical Abstract

**Figure:**
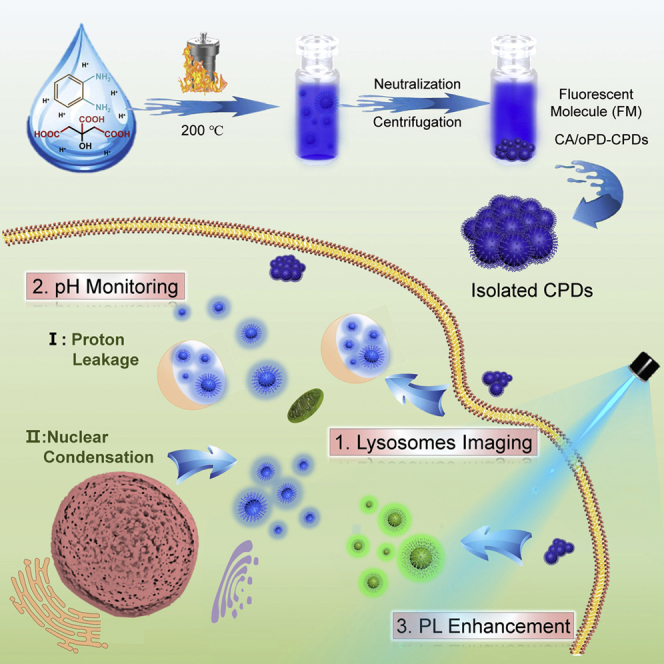

## Introduction

Protons play crucial roles in living cells during physiological and pathological processes, including intracellular protein degradation in lysosomes, homeostasis, and cell death (Gottlieb et al., 1995, Gottlieb et al., 1996, Zanke et al., 1998). Acidification is an early feature of apoptosis and cell death. The sets of proteins-enzymes related to apoptosis exclusively operate at acidic pH values. Monitoring the acidification of living cells is thus a universal approach to identify cell viability and measure the efficiency of anti-cancer drugs. To satisfy this need, the materials need to be biocompatible and able to remain in the living cells without being digested within a long period. However, some organic dyes are unable to function in acid circumstances and easily digested within hours in living cells. Thus, there is an ever-increasing demand for developing pH-responsive and extreme-pH-suitable fluorescence materials that are non-toxic and can be permanently preserved in living cells.

Compared with traditional semiconductor quantum dots (Chen et al., 2015), fluorescent carbon dots (CDs) have drawn great attentions (Baker and Baker, 2010, Yang et al., 2013, Zhang et al., 2012) due to their good biocompatibility, chemical stability, easy chemical modification, and low toxicity (Cao et al., 2013, Li et al., 2012), holding promising for use in various fields including the bioimaging (Du et al., 2019, Zhao et al., 2019, Zheng et al., 2015), optical sensing (Dong et al., 2015, Zhang et al., 2014, Zhao et al., 2015), medical diagnosis (Kong et al., 2012), catalysis (Jin et al., 2015, Li et al., 2010), and photovoltaic devices (Gupta et al., 2011). Notably, CDs can be obtained from many materials, such as polymer (Liu et al., 2012), amino acid (Jiang et al., 2012, Lu et al., 2014), protein (Liu et al., 2013), sugar (Yang et al., 2011), and some small molecules such as citric acid (Liu et al., 2013, Qu et al., 2012, Qu et al., 2014b, Zhai et al., 2012, Zhu et al., 2013), which are highly abundant on the earth. However, due to the variety of CDs, the photoluminescence (PL) mechanism and the classification of CDs are still open debates (Cayuela et al., 2016, Xia et al., 2019, Zhu et al., 2015). Generally, they can be divided into three types, including graphene quantum dots (GQDs), carbon quantum dots (CQDs), and carbon nanodots (CNDs) (Cayuela et al., 2016). Notably, those CDs from small molecules always suffered an uncompleted carbonization process, including sequential polymerization, dehydration, and carbonization. The resulting CDs consist of polymeric structures or polymer/carbon hybrid structures, reasonably regarded as carbonized polymer dots (CPDs) (Xia et al., 2019). CPDs from small molecules always contain neglected fluorescent molecules, which have been confirmed by several papers (Kasprzyk et al., 2013, Kasprzyk et al., 2015, Krysmann et al., 2012, Song et al., 2015, Zhu et al., 2016). Heretofore, efficient separation of CPDs and small molecule by-products is critical in improving related applications, yet, strategies that completely purify CPDs are always difficult to achieve.

Although CPDs have been widely applied in bioimaging field (Du et al., 2019, Zhao et al., 2019, Zheng et al., 2015), only several papers realized the selective visualization of specific organelles in cells, including nucleolus (Bao et al., 2018, Hua et al., 2018, Hua et al., 2019, Khan et al., 2018), mitochondrion (Gao et al., 2017, Hua et al., 2017), Golgi apparatus (Li et al., 2017, Yuan et al., 2017), and lysosomes (E et al., 2018, Wu et al., 2017, Zhang et al., 2018b). Lysosomes are membrane-bound cytoplasmic organelles, playing critical roles in intracellular macromolecules receiving and degradation from the secretory, endocytic, autophagic, and phagocytic pathways (Saftig and Klumperman, 2009). It is essential to distinguish lysosomes from other organelles, because the improper function of lysosomes will lead to pathological changes, such as lysosomal storage disorders, neurodegenerative diseases, and even cancer (Appelqvist et al., 2013). Different from other organelles, the pH inside of lysosomes is within an acidic range from 3.8 to 6.6 to maintain the function of the degradative enzymes inside lysosomes (Zhang et al., 2018b). Such acid values make lysosomes the most acidic organelles (Cai et al., 2017). Notably, the protons in lysosomes will leak at the early stage of apoptosis, followed by the pH increase in lysosomes and the pH decrease in the cytoplasm (Nilsson et al., 2004). After that, the intracellular pH will further decrease due to the cell nuclear condensation. Consequently, the nucleic acid and the organic acid inside the nucleus are released into the cytoplasm, serving as the indicator for the death of the cell. Nonetheless, CPDs for lysosome visualization and intracellular pH recording are still on the early stage. Yet, only several papers have been published on lysosomes imaging based on CPDs. These CPDs target lysosomes actively and only exist in lysosomes with the need of surface chemistry or afterward surface modification (E et al., 2018, Wu et al., 2017, Zhang et al., 2018b). Although numerous papers described CPDs pH sensors (E et al., 2018, Hu et al., 2014, Lu et al., 2014, Wang et al., 2019, Wu et al., 2014, Zhang et al., 2018b, Zhao et al., 2019, Zhu et al., 2013), most pH-dependent CPDs only present brighter PL emission in neutral aqueous solutions (E et al., 2018, Hu et al., 2014, Wu et al., 2014, Zhang et al., 2018b, Zhu et al., 2013) and are not suitable for acidic environment. Only a few of them possess stronger emission in acid solutions (Lu et al., 2014, Wang et al., 2019, Zhao et al., 2019). The pH sensitivity of CPDs is regarded to result from the protonation and deprotonation of the amino and carboxyl groups on the surface. Among all these papers, only several of them were applied to monitor the pH variation in cells (Wu et al., 2014), and these pH variations in cells entirely came from the buffers with different pH instead of cell metabolism.

As general bioimaging agents, CPDs possess many outstanding properties. They could stain living cells via endocytosis without triggering cell death and perform as a long-lasting imaging probe compared with organic dyes. However, severe photobleaching was found in nearly all reported CPDs and organic dyes, especially with the influence of intracellular acidification and laser irradiation of confocal microscopy. The vulnerability of these bioimaging probes strongly hindered the study of cell death, which demands the bioimaging agent to be stable with both UV light and acid. Although CPDs possess relatively better photostability than organic dyes, the unavoidable fluorescence decay is still detrimental during long-time or repeated laser exposure. Thus, developing a bioimaging agent with neglectable fluorescence loss or even enhanced fluorescence intensity over long-time laser exposure is very meaningful for long-term cell fate monitor.

In this study, we hydrothermally synthesized a new kind of CPDs from citric acid and o-phenylenediamine (named as CA/oPD-CPDs). The synthesis process includes polymerization and further controlled carbonization, forming amorphous carbon nanodots encapsulated by small polymer chains. CA/oPD-CPDs feature aggregation-dissociation adjustable properties and sensitivity to the surrounding pH, owing to the abundance of surface groups, benzene groups, carboxyl groups, and amino groups. Specifically, CA/oPD-CPDs will aggregate in neutral aqueous solution, whereas disperse in both acidic and alkaline circumstances with stable fluorescence. This property allows us to separate CA/oPD-CPDs from molecule by-products entirely via centrifugation and dialysis. After separating the small molecular fluorophore with PLQY as high as 83%, we confirm the pH-dependent PL CPDs with pretty low fluorescence at neutral pH and increased fluorescence under acidic/alkaline conditions. Benefiting from this unconventional pH-sensitive nature of CA/oPD-CPDs, we successfully managed to selectively visualize lysosomes in cells in an unprecedented real-time manner. Different from previous CPDs for lysosome imaging (E et al., 2018, Wu et al., 2017, Zhang et al., 2018b), CA/oPD-CPDs could visualize lysosomes passively regarding the acidic environment in the lysosomes. Without targeting groups, CA/oPD-CPDs possess no selectivity and spread all over the cell. Thus, they can also monitor the intracellular pH variation during the apoptosis process, which involves the pH variation in the cytoplasm. Different from other organic dyes or CPDs, which are suffering from photobleaching issue, the as-prepared CA/oPD-CPDs present a continuous fluorescence increase upon 2-h laser irradiation in cells. We confirm the fluorescence enhancement was the result of intracellular acidification. Our investigations identify promising CA/oPD-CPDs are likely essential to cell fate study and can be modulated to improve the identification of the cell viability and the efficiency of anti-cancer drugs.

## Results and Discussion

### Preparation and Characterization of Citric Acid/o-Phenylenediamine Carbonized Polymer Dots and the Fluorescent Molecule

**Figure 1:**
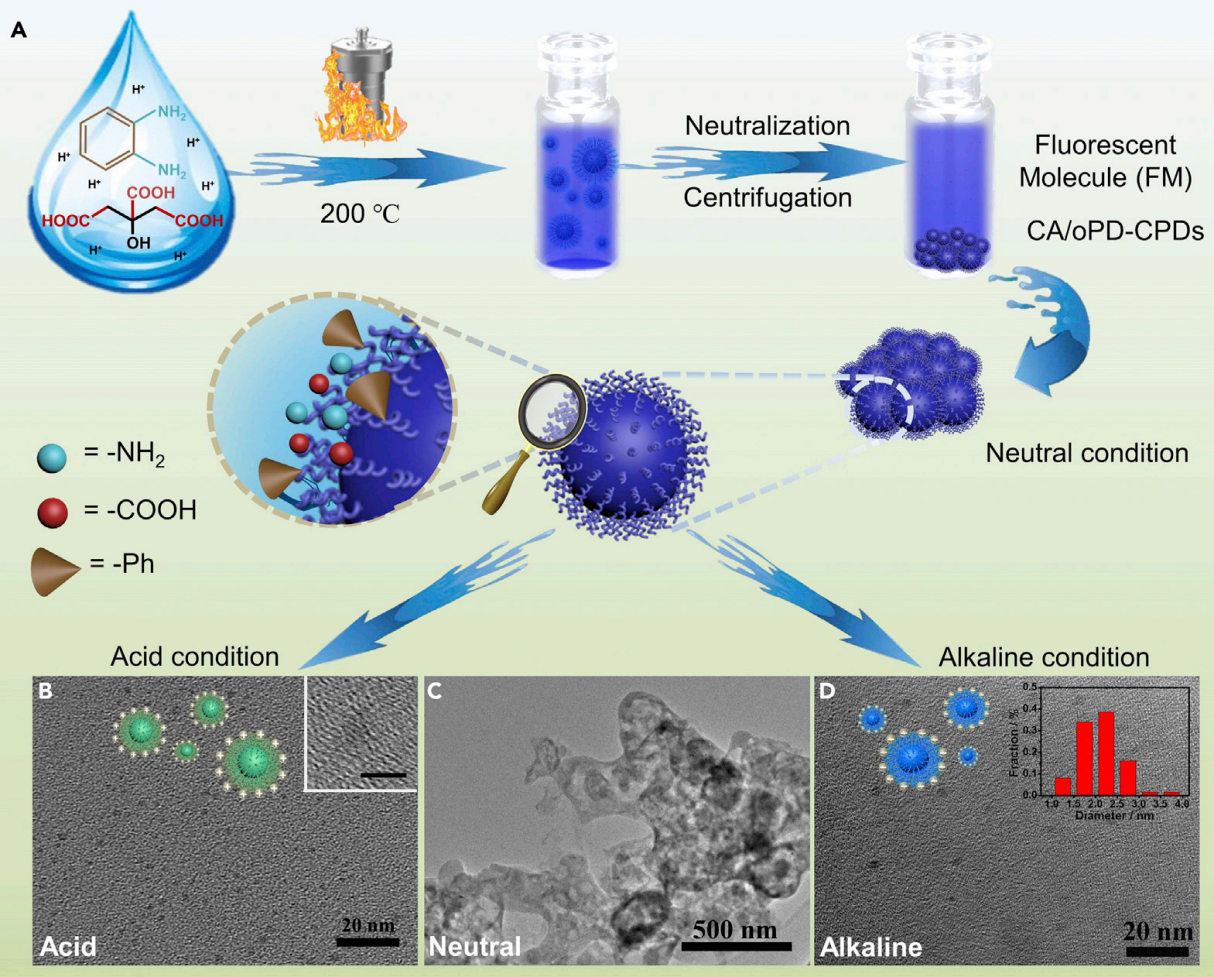
Synthesis and Morphology of CA/oPD-CPDs (A–D) (A) Preparation routes of fluorescent molecule and CA/oPD-CPDs. Morphology of CA/oPD-CPDs in (B) acid (inset is the high-resolution TEM image of CA/oPD-CPDs with a scale bar of 5 nm), (C) neutral, and (D) alkaline aqueous solutions with either HCl or NaOH (inset is the size distribution of CA/oPD-CPDs).

The basic procedures to synthesize CA/oPD-CPDs include the decomposition and pyrolysis of citric acid and o-phenylenediamine at 200°C for 8 h. After being neutralized and centrifuged, the supernatant (fluorescent molecule, FM) and sediment (CA/oPD-CPDs) were collected and purified, respectively, as illustrated in Figure 1A. In the UV-vis absorption spectra of FM and CA/oPD-CPDs, peaks at 240 and 277 nm are presumably attributed to π-π∗ transition, whereas the peak at 302 nm in FM is supposedly the result of n-π∗ transition of C=O. The wide peak in CA/oPD-CPDs from 300 to 450 nm results from the complex carbon core (Figure S1A). We confirmed the detailed structures of both FM and CA/oPD-CPDs as follows.

We initially investigated the chemical compositions of CA/oPD-CPDs. In the FTIR analysis of CA/oPD-CPDs, the following groups were observed: the stretching vibrations of O-H/N-H at 3,436 cm^−1^ and 3,197 cm^−1^, the stretching vibrations of C-H at 2,919 cm^−1^, the peak at 1,643 cm^−1^ assigned to carboxyl (COOH) group, whereas the stretching vibrations of C-N at 1,385 cm^−1^, the peaks at 1,157 cm^−1^ and 1,076 cm^−1^ corresponded to C-O and N-H groups (Figure S1B) (Bao et al., 2018, Lu et al., 2018). These functional groups result from reactants and could be further confirmed by X-ray photoelectron spectroscopy (XPS) spectra. The XPS spectra of CA/oPD-CPDs comprised three peaks at 285.0, 400.0, and 532.0 eV, which were attributed to C_1s_, N_1s_, and O_1s_, respectively (Figure S2). The C_1s_ spectrum of CA/oPD-CPDs indicates the presence of three types of carbon bonds: sp^2^ carbon (C-C/C=C) at 284.78 eV, sp^3^ carbon (C-N/C-O) at 286.3 eV, and oxidized carbon (C=O) at 288.34 eV (Qu et al., 2014a). The N_1s_ spectrum of CA/oPD-CPDs shows two types of N bonds: C-N-C (398.4 eV) and N-(C)_3_ (400.25 eV) (Qu et al., 2014b). The O_1s_ spectrum of CA/oPD-CPDs shows two types of O bonds: C=O (531.21 eV) and C-OH or C-O-C (533.1 eV) (Feng et al., 2017). Moreover, No D/G band was observed in the Raman spectrum of CA/oPD-CPDs (Figure S3), which further confirmed the amorphous feature of CA/oPD-CPDs. Further more, the elemental composition of CA/oPD-CPDs was measured by elemental analysis, and the elemental content of C 66.97%, H 4.57%, N 15.17%, and O (calculated) 13.29% are provided (Table S1).

Although the existence of benzene groups in CA/oPD-CPDs increased the hydrophobic degree of the whole nanoparticles, the carboxyl groups and amino groups endowed CA/oPD-CPDs with hydrophilic properties in proper pH aqueous solution. As a result, CA/oPD-CPDs are soluble in acid and basic conditions but aggregate in the neutral condition, benefitting the separation of CA/oPD-CPDs from small molecules. As shown in Figure 1D, the diameters of CA/oPD-CPDs were mainly distributed in the range of 1–4 nm with an average diameter of 2.1 nm without lattice structure under high-resolution TEM (inset Figure 1B). At acid or alkaline conditions, CA/oPD-CPDs were either with positive (protonation) or negative (deprotonation) charges and dispersed in the solution as small nanoparticles as shown in Figures 1B and 1D. At neutral condition, the whole CA/oPD-CPDs behave as electrically neutral particles. With the existence of benzene groups, the hydrophobic degree of the whole particles increased and further aggregated in the solution as sediment (Figure 1C). The transformation of CA/oPD-CPDs between well-dispersing nanoparticles and sediment was also illustrated in Figure 1A. Additionally, the hydrophobic groups on CA/oPD-CPDs featured an excellent solubility in organic solvents (Figure S4). The fluorescence intensity increased dramatically compared with that in neutral pH aqueous solution, indicating CA/oPD-CPDs could be well dispersed in the organic phase.

We next aimed to prove the chemical structure of FM. For morphology testing, no nanodots were observed under TEM in FM samples. To analyze the chemical composition of FM, a liquid chromatography-mass spectrometer was used to identify the molecular weight of FM. The main molecular weight was focused on 202, with different retention time. We confirmed that there were a lot of isomerides with the same molecular weight 202, which are the derivative of 1-oxo-1,5-dihydropyrido[1,2-a] benzimidazole-3-carboxylic acid (Figure S5) (Kasprzyk et al., 2015). We also proved that the emission center of CA/oPD-CPDs was covalently FM binding in the CA/oPD-CPDs.

### Optical Properties of Citric Acid/o-Phenylenediamine Carbonized Polymer Dots and the Fluorescent Molecule

**Figure 2:**
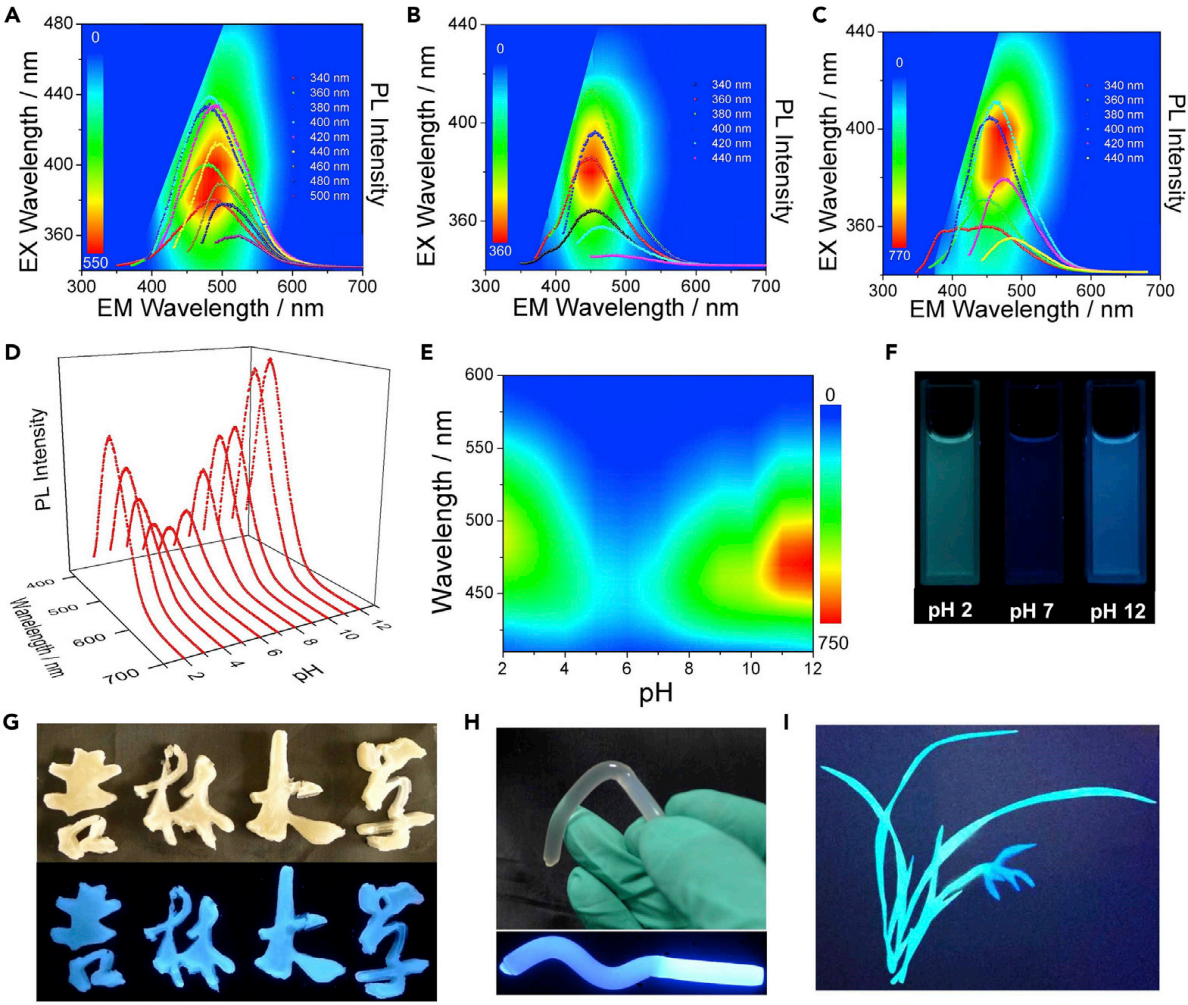
Fluorescence Behavior of CA/oPD-CPDs (A–C) PL spectra with different excitation wavelengths of CA/oPD-CPDs and corresponding excitation-emission matrix in (A) pH 2, (B) pH 7, and (C) pH 12 aqueous solutions with either HCl or NaOH. (D) The PL spectra of CA/oPD-CPDs excited with 380 nm at different pH values. (E) Emission-pH matrix of the same concentration CA/oPD-CPDs aqueous solution with different pH. (F) Digital photos of CA/oPD-CPDs in pH 2, pH 7, and pH 12 aqueous solutions with either HCl or NaOH. (G and H) Composites of cellulose and CA/oPD-CPDs under natural light and UV light. (G) Composites with urea and LiOH. (H) The stiff and brighter part of the composites after removing extra urea and LiOH. (I) Hand drawing obtained from CA/oPD-CPDs in HCl solution and FM in neutral solution. See also Tables S2 and S3 and Figures S6–S9.

We then evaluated the fluorescence properties of FM and CA/oPD-CPDs. Because CA/oPD-CPDs and FM are sensitive to pH, the fluorescence spectra of CA/oPD-CPDs and FM at different pH conditions were provided in Figures 2A–2C and S6. The emission peak positions of CA/oPD-CPDs and FM were different at different pH conditions. As depicted in Figure S6, the emission peaks of FM were located at 462, 432, and 460 nm at pH 3, 7, and 12, respectively. Notably, the emission peak presents redshift in both acid and base conditions. Similar to FM, the emission peaks of CA/oPD-CPDs were 482, 450, and 470 nm at pH 2, 7, and 12, respectively. Redshifts were also observed in CA/oPD-CPDs, which further indicates the same fluorescence center in both FM and CA/oPD-CPDs. Noticeably, even though the same fluorescence groups were presumably responsible for the PL origin of both FM and CA/oPD-CPDs, excitation dependent behavior was obviously observed in CA/oPD-CPDs, which was not found in FM. These results indicate that “carbon core” is one of the reasons for excitation dependent behavior. To further analyze this pH-dependent behavior, the fluorescence spectra were analyzed (Figure S7). After normalization, it is clear that the emission peaks shift from greenish to blue region from pH 2 to pH 7 and shift back from pH 7 to pH 12. In this circumstance, redshift was also observed in alkaline conditions (Figure S7). We suppose it could be the result of protonation and reversible chemical reactions, such as keto-enol tautomerism (Figure S8). Notably, this reversible reaction is promoted both in acid and alkaline conditions. The similar fluorescence behavior was also observed in FM (Figure S9).

Besides different fluorescence peak positions, the fluorescence intensities also varied with different pH conditions. For FM, the highest fluorescence intensity was observed at the neutral condition, whereas for CA/oPD-CPDs, the fluorescence intensities were much higher at both acid and alkaline conditions (Figures 2D and 2E). This phenomenon is obviously different from reported CPDs (Bao et al., 2018, Kasprzyk et al., 2013) and makes it possible to apply CA/oPD-CPDs in cell death studies and other extreme pH situations. We believe it is because CA/oPD-CPDs aggregated at neutral condition, and the aggregated CA/oPD-CPDs further induced the self-quenching effect, which decreased the fluorescence intensity. This theory was further proved by the CA/oPD-CPDs and cellulose composites.

Due to the superior alkali resistance ability of the synthesized CA/oPD-CPDs, it is the best candidate for the fabrication of cellulose composites, which involves massive LiOH and urea (Wang et al., 2014). Figures 2G and 2H described the fluorescence of the composites remained bright even under extremely alkaline condition, and even increased after removing excess urea and LiOH (Figure 2H). At alkali condition, the enhanced dispersion of CA/oPD-CPDs in the composites retains the bright blue fluorescence. Although even the composite became neutral after removing urea and LiOH, the fluorescence of CA/oPD-CPDs was still witnessed owing to the restriction of the polymer network. This further proved that the aggregation of CA/oPD-CPDs at neutral condition was responsible for the quenching of fluorescence. Furthermore, the pH-dependent emission property of CA/oPD-CPDs and FM was additionally applied as fluorescence ink (Figure 2I).

To further investigate the fluorescence differences between FM and CA/oPD-CPDs, we tested the fluorescence lifetime of FM and CA/oPD-CPDs. As shown in Table S2, FM possessed a single lifetime, 9.42 ns at the neutral condition and 10.53 ns at alkaline condition, respectively. Although CA/oPD-CPDs showed two lifetimes, 2.5 ns related to the fluorescence from carbon core and 9.0 ns corresponding to fluorophores covalently anchored on the surface of carbon core. Noticeably, a new lifetime, 4.89 ns, appeared in the case of FM at acid condition, which could result from the aggregation of small molecules. At acid condition, the fluorescence spectra of FM presented slightly excitation-dependent behavior (Figure S6A), and the quantum yield of FM decreased to 43%, much lower than 83% at alkaline and neutral conditions (Table S3). Conclusively, we infer there are some aggregations of FM at acid condition. These aggregations at acid conditions change the surrounding environment and create a carbon-core similar situation, leading to the excitation-dependent behavior and the decreasing of PLQY. Analogously, this phenomenon was also found in other fluorescent organic molecules (Wang et al., 2015). As fluorescent materials, outstanding photostability is crucial for their further applications. After 8 h of continuous UV exposure, the fluorescence of the FM decreased to 32.58% (Figure S10). On contrast, 64.62% fluorescence of the CA/oPD-CPDs was preserved. The carbon core was supposed to partially protect the emission center, and even some fluorophores bonded covalently to the core was quenched by UV light (Zhai et al., 2012).

Besides UV exposure, cations could also influence the fluorescence intensity. We thus investigated the influence of different cations on CA/oPD-CPDs. To rule out the disturbance of cations to the pH of solutions, disodium hydrogen phosphate/citric acid buffer with pH 7.0 was chosen as the model solution. Herein, common metal ions in cell media, such as Na^+^, K^+^, Mg^2+^, Ca^2+^, Zn^2+^, Al^3+^, Ni^2+^, Cu^2+^, and Fe^3+^ at a concentration of 10^−2^mol L^−1^ were added in CA/oPD-CPDs solutions (10 μg mL^−1^; Figure S11). Similar to typical carbon nanodots, Fe^3+^ ion also had the obvious quenching effect on CA/oPD-CPDs (Zhu et al., 2013). For Ca^2+^, Zn^2+^, and Cu^2+^ ions, slight fluorescence variations can be attributed to the interactions between carboxylic groups and metal ions.

### Selective Visualization of Lysosomes

**Figure 3:**
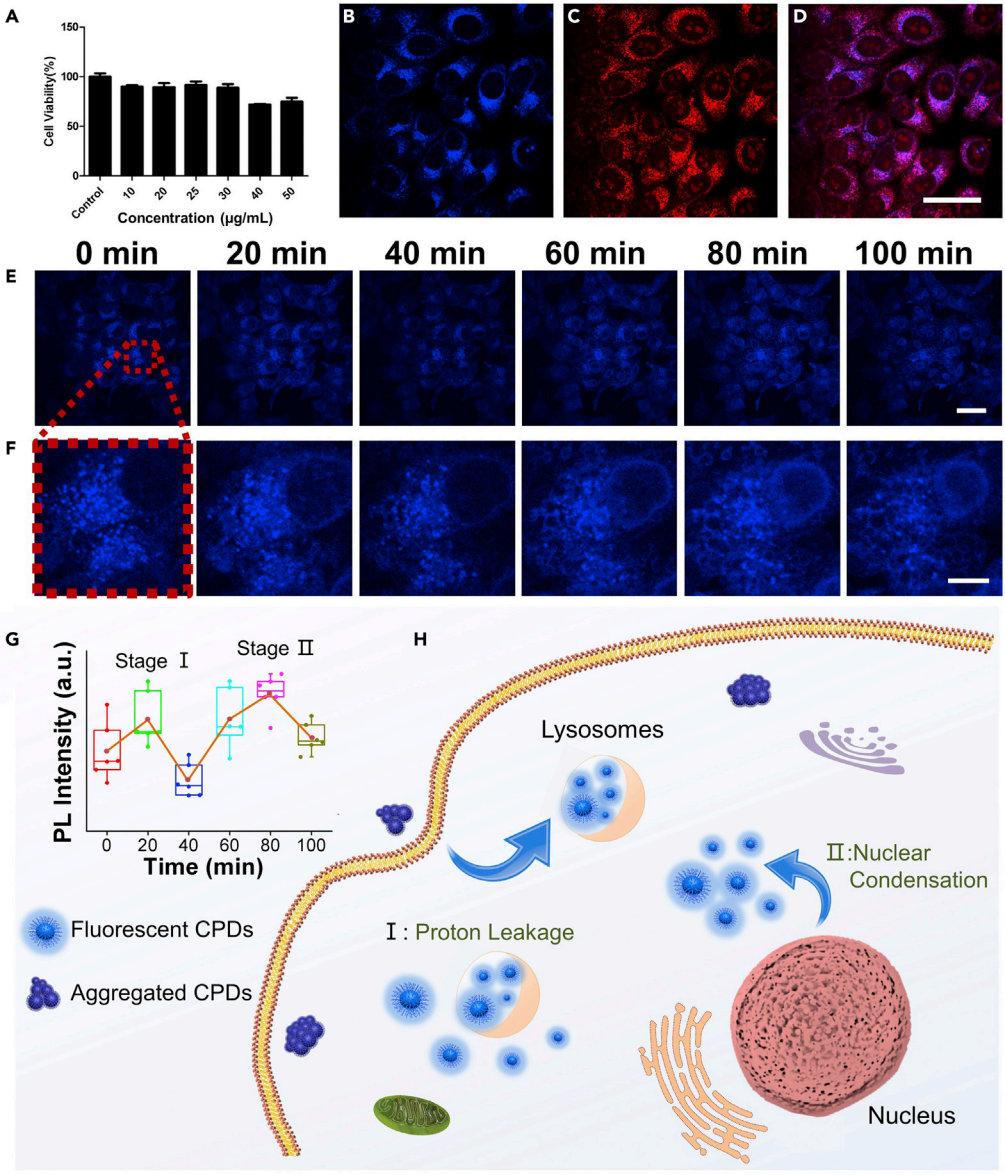
CA/oPD-CPDs for Lysosomes Imaging and Intracellular pH Monitoring (A) Cytotoxicity assay of CA/oPD-CPDs on A549 cells (n = 3, Mean ± SD). (B–D) Confocal fluorescence images of A549 cells co-stained with CA/oPD-CPDs and LysoTracker Red excited with (B) 405 nm and (C) 536 nm and (D) overlayed images of (B) and (C) (Scale bar: 40 μm). (E and F) Real-time intracellular pH monitoring in living A549 cells during the apoptosis process. (E) Confocal fluorescence images of A549 cells incubated with CA/oPD-CPDs during apoptosis process on a large scale (Scale bar: 40 μm). (F) Magnified confocal fluorescence images of single A549 cell during the apoptosis (Scale bar: 10 μm). (G) PL intensity variation of CA/oPD-CPDs in living cells at different apoptosis stage (n = 6, Mean ± SD). (H) The intracellular trafficking and transformation of CA/oPD-CPDs during apoptosis. See also Figures S12 and S13.

As a tracking probe in biological events, cytotoxicity of CA/oPD-CPDs was first evaluated with MTT assay in human lung carcinoma (A549) cells. As shown in Figure 3A, when the concentration of CA/oPD-CPDs was 25 μg/mL, a sufficient concentration dosage for bioimaging, the cell viability of A549 was still 91.8%. When we further increased the incubation concentration to 50 μg/mL, the cell viability of A549 slightly decreased to 74.9%. These results indicated that the synthesized CA/oPD-CPDs were biocompatible and with extremely low toxicity within the concentration range for bioimaging, laying a solid foundation for the further application of CA/oPD-CPDs in biological fields.

We then examined whether the CA/oPD-CPDs can be adopted for lysosomes visualizing and tracking. The A549 cells were firstly incubated with CA/oPD-CPDs with a concentration of 25 μg/mL for 24 h. Then the cells were incubated with 100 nM LysoTracker Red DND99, a lysosome-targeting dye, to co-stain the lysosomes in cells. As shown in Figures 3B–3D, the blue fluorescence from CA/oPD-CPDs and the red fluorescence from LysoTracker Red overlapped with a high Pearson's correlation coefficient (a measure of the linear association of two variables, herein, fluorescence from CA/oPD-CPDs and those from LysoTracker Red) of 0.88, exceeding that of most lysosome-targeting nanoparticles. Lysosomes, acidic organelles with a pH around 4.8, play an important role in intracellular macromolecules degradation. Benefiting from the acidophilous property of CA/oPD-CPDs, we managed to visualize lysosomes in living cells passively without the involvement of additional lysosome-targeting molecular structure. Although CA/oPD-CPDs possess no selectivity in cells and spread over the whole cell, only the CA/oPD-CPDs in the lysosomes presented bright fluorescence due to the appropriate pH inside. Most CA/oPD-CPDs in the cytoplasm aggregated and presented extremely weak fluorescence. Thus, selective visualization of lysosomes was achieved with our pH-sensitive CA/oPD-CPDs. Because most pH-sensitive CPDs present weak fluorescence in acidic solution, the weak fluorescence from lysosomes will be covered by the strong fluorescence from cytoplasm and make it unable to visualize lysosomes without targeting groups. To the best of our knowledge, this is the first report of passive targeting CPDs applied in selective visualization of organelles. Moreover, compared with organic dyes with photostability issue, the CA/oPD-CPDs are able to preserve in living cells for 24 h without being degraded or causing cell death, which presents a possibility for long-time tracking of living cells.

### Real-Time Intracellular pH Monitoring During Apoptosis

Besides selective visualization of lysosomes, CA/oPD-CPDs could also be applied for real-time cell pH monitoring during apoptosis. As discussed earlier, CA/oPD-CPDs possess no targeting groups and spread over the cytoplasm. CA/oPD-CPDs possess extremely weak fluorescence in the healthy living cells owing to the neutral physiological pH value. Only the well-dispersed CA/oPD-CPDs in the lysosomes present bright blue emission. Thus, the aggregated CA/oPD-CPDs in living cells could be used to monitor the intracellular pH change during apoptosis. After incubation for 24 h with CA/oPD-CPDs, a strong blue fluorescence from CA/oPD-CPDs in the lysosomes was observed as shown in Figure 3E. We then used dexamethasone (a chemotherapeutic agent) to induce apoptosis of living cells. Without the addition of dexamethasone, the fluorescence intensity from the control group (CA/oPD-CPDs only) continuously decreased due to photobleaching under UV light (Figure S12). After four times of exposure, the original fluorescence intensity decreased a lot (Figure S13). For the experiment group (CA/oPD-CPDs with the addition of dexamethasone), the fluorescence intensity from the whole cell involves two stages of fluorescence increasing, as shown in Figure 3E. After 20-min treatment by dexamethasone, the first stage originated from proton leakage from lysosomes. The fluorescence intensity slightly increased (Figure 3G). In the magnified image (Figure 3F) the contour of lysosomes became fuzzy at the first stage. Due to proton leakage, the pH inside the lysosomes increased and the fluorescence of CA/oPD-CPDs in the lysosomes was suppressed. However, the leaked proton illuminated the CA/oPD-CPDs in the cytoplasm around the lysosomes. Thus, the contour of lysosomes became unclear and the fluorescence from the whole cell slightly increased even under the influence of photobleaching. After 40 min, the fluorescence intensity decreased due to photobleaching. During the apoptosis process, proton leakage takes place firstly, following by the second stage, nuclear condensation. The nuclear acid and other organic acids were leaked through the nuclear envelope, further decreasing the pH in the cytoplasm. Thus, the aggregated CA/oPD-CPDs in the cytoplasm were illuminated with the acidic materials around nuclear envelope after the addition of dexamethasone for 60 min. As shown in Figure 3G, the fluorescence intensity further increased for whole cells indicating the decrease of intracellular pH. Moreover, the nuclear envelop contour was also illuminated and getting condensed during the apoptosis process. For other pH-sensitive CPDs, the fluorescence intensity always decreases during apoptosis, similar to the tendency caused by photobleaching, which makes it unapparent to monitor the intracellular pH variation without a control group. Different from other reports, the fluorescence intensity of the synthesized CA/oPD-CPDs increased obviously during apoptosis even with the influence of photobleaching. Benefitting from their aggregation-dissociation induced PL behavior, CA/oPD-CPDs achieved persistently recording of intracellular pH during apoptosis even with partial photobleaching issue.

### Laser-Induced Fluorescence Enhancement for Living Cells

**Figure 4:**
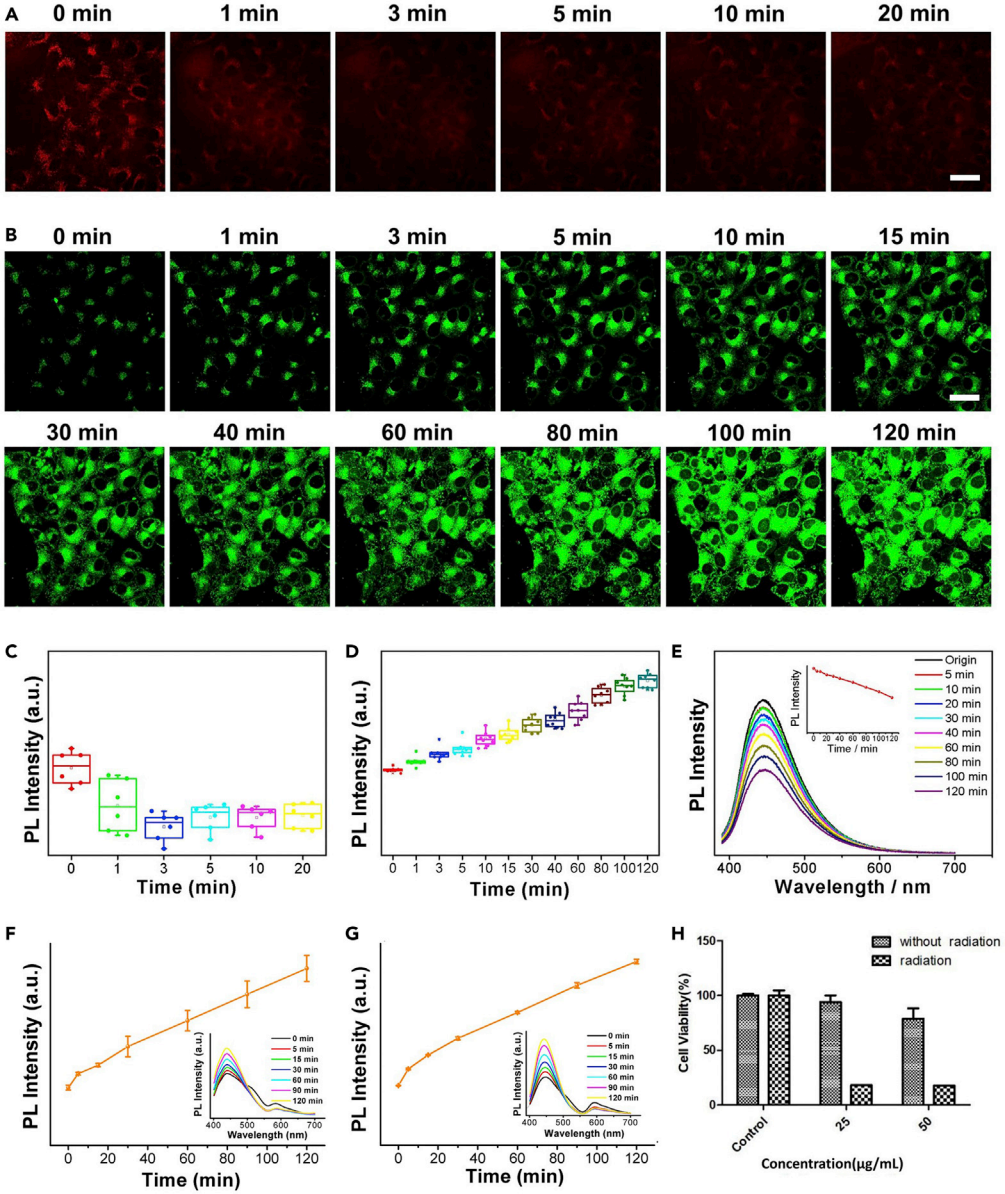
Laser-Induced Fluorescence Enhancement of CA/oPD-CPDs in Living Cells (A and C) A549 cells stained with Lyso-Tracker Red continuously exposed for different periods under 536 nm laser irradiation. (A) confocal fluorescence images and (C) corresponding PL intensity variation (Scale bar: 40 µm; n=6, Mean±SD). (B and D) A549 cells stained with CA/oPD-CPDs continuously exposed for different periods under 488 nm laser irradiation. (B) confocal fluorescence images and (D) corresponding PL intensity variation (Scale bar: 40 µm; n=8, Mean±SD). (E) PL spectra and intensity variation of CA/oPD-CPDs aqueous solution for different exposure periods under 488 nm in neutral aqueous solution. (F and G) PL intensity variation and spectra of living (F) A549 cells (n = 3, Mean ± SD) and (G) L929 cells suspension (n = 3, Mean ± SD) incubated with CA/oPD-CPDs for different exposure periods under 488 nm (tested with fluorescence spectroscopy). (H) Cytotoxicity assay on A549 cells of CA/oPD-CPDs with and without 2-h 15 W blue light exposure (n = 3, Mean ± SD). See also Figures S14–S16.

Besides selective visualization of lysosomes and intracellular pH monitoring, another fascinating feature of our CA/oPD-CPDs is the laser-induced fluorescence enhancement. Fluorescence loss under laser irradiation is hindering the development of most bioimaging agents, particularly under the laser confocal microscopy. LysoTracker Red is a representative commercial lysosomal dye, whose fluorescence intensity quickly decreased within 3-min exposure (Figures 4A and 4C). In contrast, the synthesized CA/oPD-CPDs presented a continuous fluorescence increase under laser irradiation in living cells within 2 h (Figures 4B and 4D, A549 cells (human non-small cell lung cancer cell lines, cancer cells) were cultured with CA/oPD-CPDs in advance for 24 h to promote efficient endocytosis). To understand this phenomenon, several control experiments were conducted. For CA/oPD-CPDs aqueous solution, no fluorescence increase was observed with blue-light exposure (Figure 4E). When living A549 cells with CA/oPD-CPDs were exposed under blue light *in vitro* with different times, the fluorescence intensity increased with longer exposure time (Figure 4F). To verify the universality of this fluorescence enhancement, L929 cells (mouse-derived fibroblast cells, normal cells) were also applied. As shown in Figure S14, L929 cells also presented a continuous fluorescence enhancement under laser confocal microscopy. The fluorescence enhancement was also observed with fluorescence spectroscopy (Figure 4G). Because L929 cells are normal cells, they are more vulnerable than A549 cells. Under the same blue light exposure, the cytoplasm of L929 cells turned acid faster than A549 cells, leading to a higher fluorescence enhancement (insets of Figures 4F and 4G) under the same exposure time. Thus, we supposed that the CA/oPD-CPDs with blue light could induce slowly death of living cells by reactive oxygen species. Photodynamic therapy (PDT) can cause irreversible damage to cells through reactive oxygen species (ROS) that are generated by photosensitizers (PS). The death of cells will inevitably cause the acidification in cytoplasm (Gottlieb et al., 1995, Gottlieb et al., 1996, Zanke et al., 1998). Among all the applications of CPDs, unmodified CPDs as photosensitizers applied in photodynamic therapy are still uncommon (Du et al., 2019, Lu et al., 2019, Zhang et al., 2018a). Herein, the generated ROS from CA/oPD-CPDs under blue light caused cell damage, leading to the acidification of cytoplasm, and further increased the fluorescence of CA/oPD-CPDs in cells. To prove this theory, the cytotoxicity of CA/oPD-CPDs with blue light was tested. When the concentration of CA/oPD-CPDs was 25 μg/mL, the cell viability of A549 was 21% with 2-h 15 W blue light exposure but 91% without blue light exposure (Figure 4H). Then, the DCFH-DA fluorescent probe was applied to confirm the existence of ROS. Fluorescence intensity from DCFH-DA with CA/oPD-CPDs under blue light increased dramatically compared with the control group (Figure S15), which confirmed that CA/oPD-CPDs could generate ROS under blue light exposure. To further confirm this assumption, the fluorescence behavior of fixed cells (dead cells) under laser confocal microscopy was tested. As shown in Figure S16, the fluorescence intensity from fixed A549 cells expectedly decreased with longer laser exposure time. Based on these experiments, we believe that the acidification of living cells under laser exposure is responsible for the fluorescence enhancement of CA/oPD-CPDs. Additionally, the synthesized CA/oPD-CPDs also present a potential utilization as photodynamic agents for cancer therapy.

### Conclusion

In summary, we synthesized a kind of CA/oPD-CPDs with unconventional pH sensitivity from citric acid and o-phenylenediamine via a hydrothermal process. We not only confirmed the co-existence of fluorescent molecules and CA/oPD-CPDs but also separated them with a simple method and fully investigated their differences. The synthesized CA/oPD-CPDs presented bright fluorescence at acidic and alkaline conditions but weak fluorescence in the neutral environment. Benefitting from this property, we achieved passive lysosomes visualization. Further more, the aggregated dormient CA/oPD-CPDs in the cytoplasm were able to monitor real-time intracellular pH variation during apoptosis of living cells including the proton leakage and cell nuclear condensation. Moreover, as a bioimaging agent, the CA/oPD-CPDs presented a continuous fluorescence enhancement under laser irradiation, which laid a solid foundation for single-cell long-term tracking. Developing unconventional pH-responsive CPDs that are non-toxic and can be preserved in living cells within a long period is highly meaningful and provides useful modality on the study of cell fate, cell viability, and the efficiency of anti-cancer drugs.

### Limitations of the Study

Although the synthesized CA/oPD-CPDs are sensitive to pH and could track the intracellular pH variation, the UV-induced photobleaching is still hindering the quantification of specific intracellular pH.

## Methods

All methods can be found in the accompanying Transparent Methods supplemental file.

## Supplemental Information

Document S1. Transparent Methods, Figures S1–S16, and Tables S1–S3
