# Supplementary material for: Self-Enhanced Carbonized Polymer Dots for Selective Visualization of Lysosomes and Real-Time Apoptosis Monitoring
Source: iScience. 2020 Mar 13;23(4):100982. doi: 10.1016/j.isci.2020.100982 (PMC7113624; doi:10.1016/j.isci.2020.100982)
Supplement: Document S1. Transparent Methods, Figures S1–S16, and Tables S1–S3 [file mmc1.pdf]

iScience, Volume *23*

Supplemental Information

Self-Enhanced Carbonized Polymer Dots for Selective Visualization of Lysosomes and Real-Time Apoptosis Monitoring

Xiaohuan Zhao, Jing Li, Dongning Liu, Mingxi Yang, Wenjing Wang, Shoujun Zhu, and Bai Yang

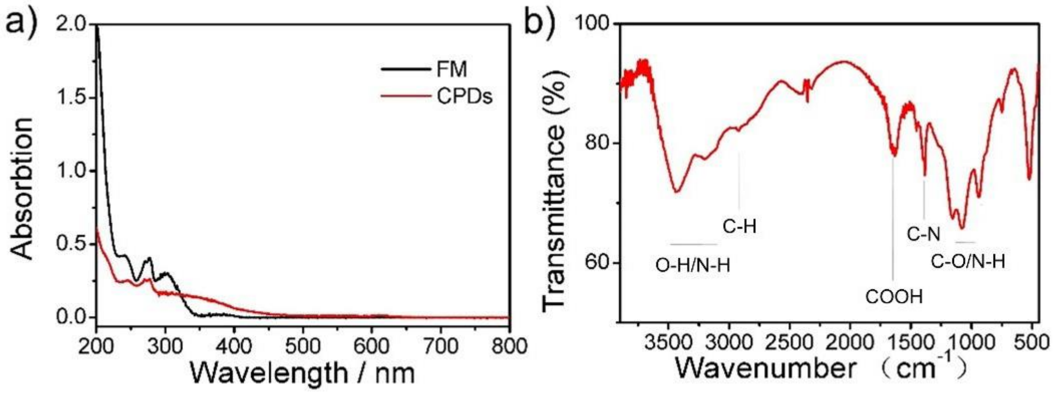

Figure S1. a) UV-vis absorption of FM and CA/oPD-CPDs in neutral aqueous solution. b) FTIR spectrum of CA/oPD-CPDs powder. Related to Figure 1.

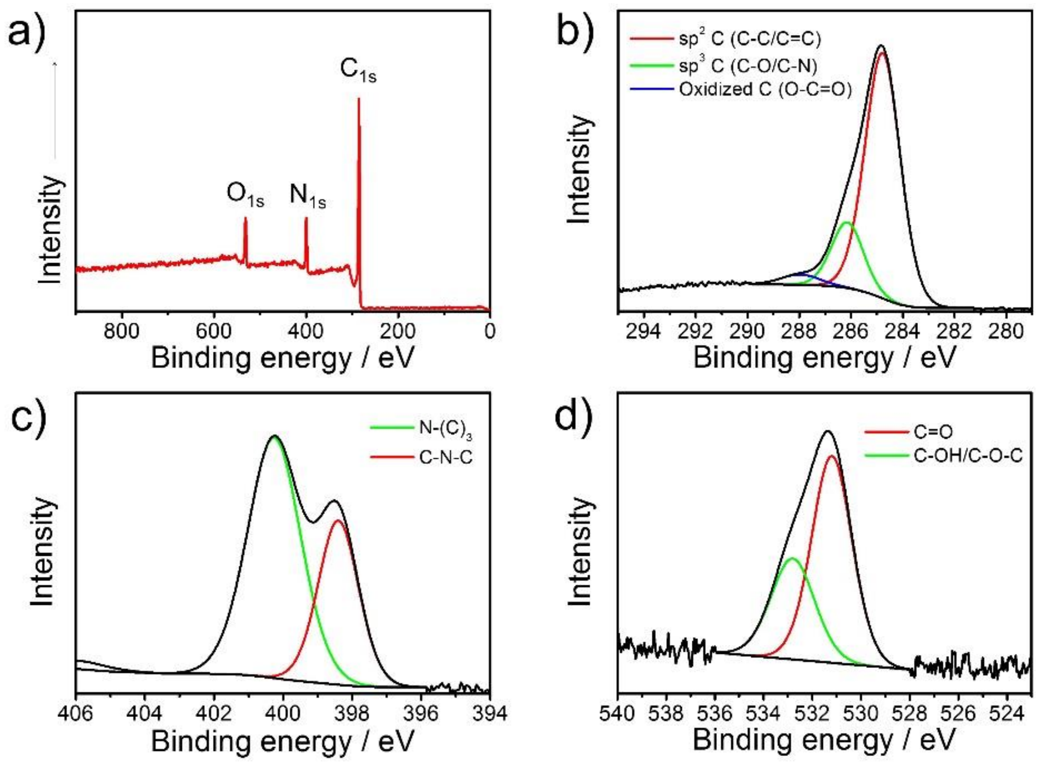

Figure S2. XPS spectra of CA/oPD-CPDs, which were tested with CA/oPD-CPDs powder on glass. Related to Figure 1.

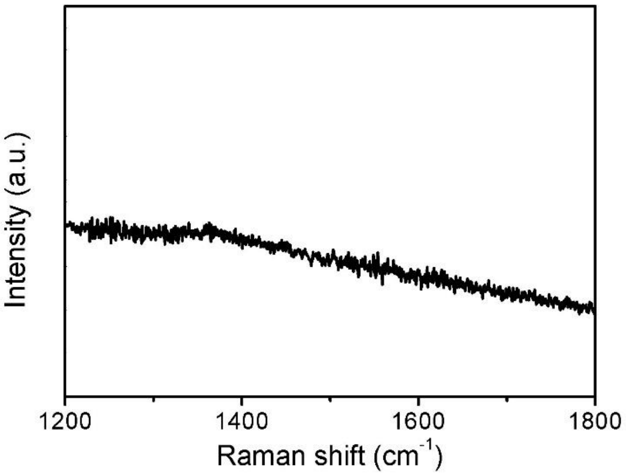

Figure S3. The Raman spectrum of CA/oPD-CPDs (tested with CA/oPD-CPDs powder on glass). Related to Figure 1.

Table S1. Elemental analysis of CA/oPD-CPDs which was tested with CA/oPD-CPDs powder. Related to Figure 1.

Element	C(%)	N(%)	H(%)	O (Calculated)(%)
CA/oPD-CPDs	66.97%	15.17%	4.57%	13.29%

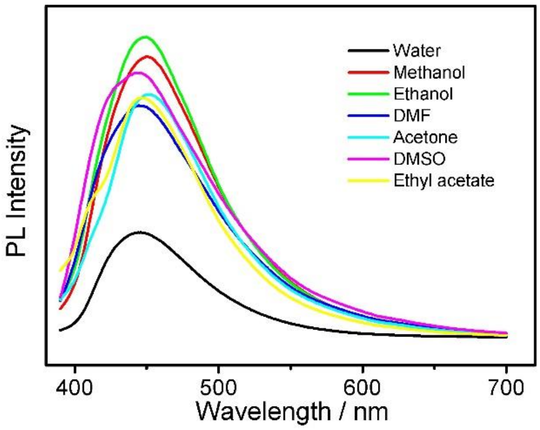

Figure S4. Fluorescence spectra of CA/oPD-CPDs in neutral pH aqueous solution and other different organic solvents. Related to Figure 1.

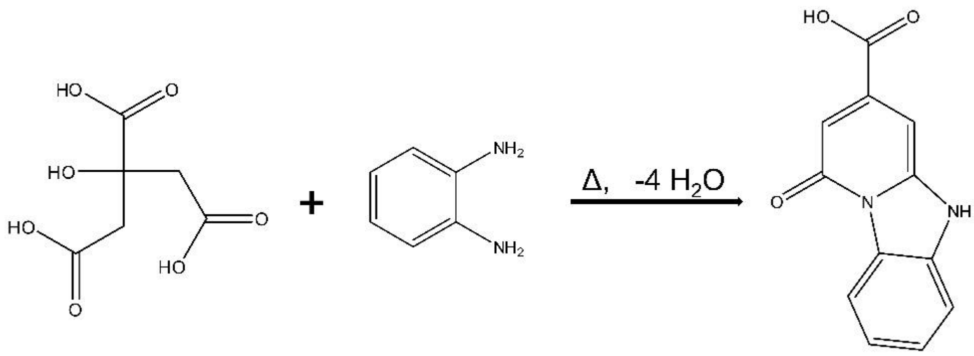

Figure S5. Scheme of the primary reaction of citric acid and o-phenylenediamine. Related to Figure 1.

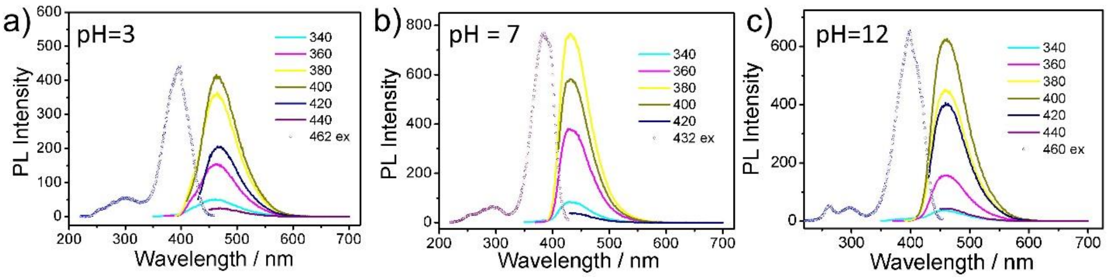

Figure S6. PL spectra of FM with different excitation wavelengths in a) pH 3, b) pH 7, and c) pH 12 aqueous solutions with either HCl or NaOH. Related to Figure 2.

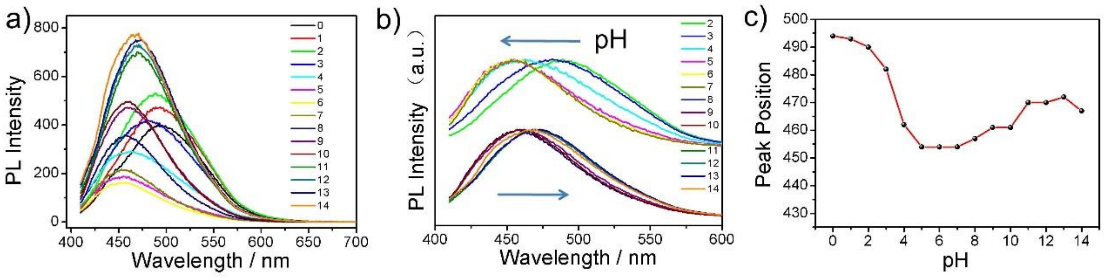

Figure S7. a) The PL spectra of CA/oPD-CPDs excited with 380 nm at different pH conditions and b) the normalized PL spectra. c) PL peak positions of CA/oPD-CPDs at different pH conditions. The PL spectra were tested in different pH aqueous solutions with HCl, NaOH or citric acid/disodium hydrogen phosphate buffers. Related to Figure 2.

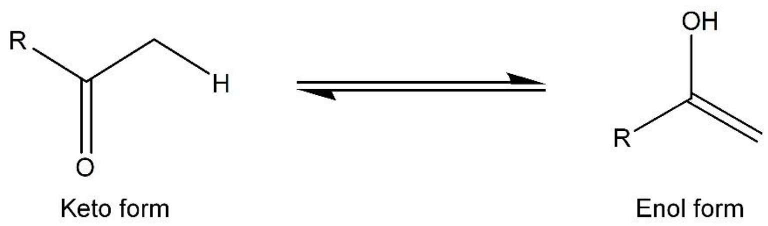

Figure S8. Scheme of the Keto-Enol Tautomerism. Related to Figure 2.

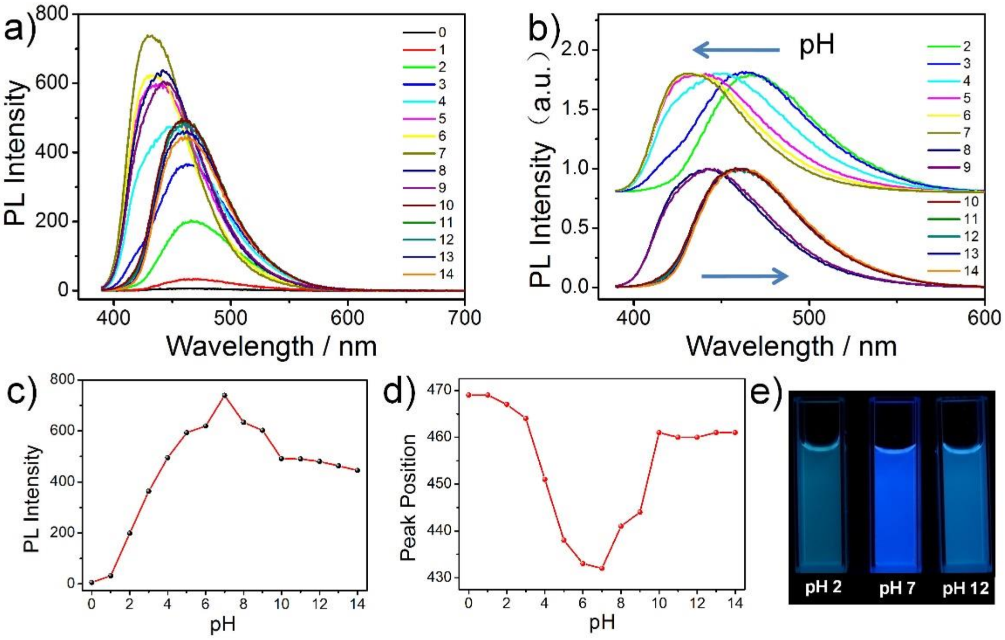

Figure S9. a) The PL spectra of FM at different pH conditions. b) Normalized PL spectra of FM. c) PL intensities and d) PL peak positions of FM at different pH conditions. e) Digital photos of FM solutions at pH 2, pH 7, and pH 12. The PL spectra were tested in different pH aqueous solutions with HCl, NaOH or citric acid/disodium hydrogen phosphate buffers. Related to Figure 2.

Table S2. The Fluorescence lifetime of FM and CA/oPD-CPDs (tested in different pH aqueous solutions with either HCl or NaOH). Related to Figure 2.

	**pH 2**		**pH 7**		**pH 12**	
**FM**	4.89 ns	10.64 ns	9.42 ns		10.53 ns	
	12.36%	87.64%	100%		100%	
	9.93 ns		9.42 ns		10.53 ns	
**CA/oPD-CPDs**	2.39 ns	8.64 ns	3.13 ns	9.14 ns	2.53 ns	9.31 ns
	23.66%	76.34%	20.57%	79.43%	26.87%	73.13 %
	7.16 ns		7.90 ns		7.49 ns	

Table S3. PLQY of FM and CA/oPD-CPDs at pH 2, pH 7, pH 12. Related to Figure 2.

pH	2	7	12
FM	43%	83%	83%
CA/oPD-CPDs	19.9%	5.79%	20.16%

Note: CA/oPD-CPDs and Fluorescent Molecule were dispersed as very dilute solutions in pure water (pH 7), water with HCl (pH 2), and water with NaOH (pH 12), respectively. The absolute quantum yields were tested with an excitation wavelength of 380 nm.

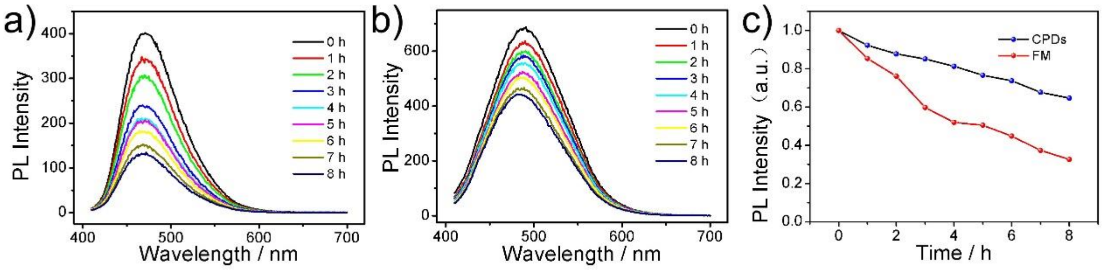

Figure S10. PL spectra of a) FM and b) CA/oPD-CPDs at pH 2 aqueous solutions with HCl under continuous UV exposure. c) PL intensity decreasing tendency of FM and CA/oPD-CPDs. Related to Figure 2.

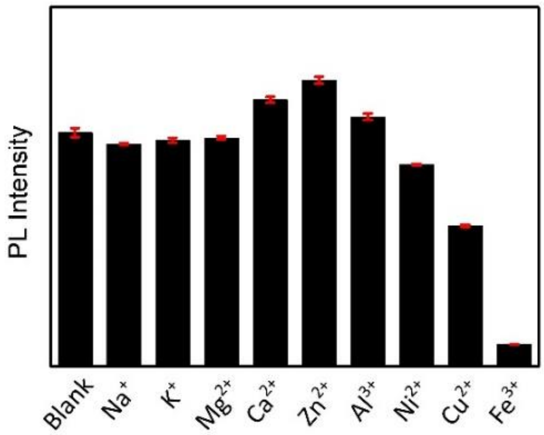

Figure S11. Comparison of fluorescence intensities of CA/oPD-CPDs (10 μg mL^{-1}) after the addition of different metal ions (10^{-2} mol L^{-1}). (Due to the pH sensitivity of CA/oPD-CPDs, the PL intensities were tested in pH 7 citric acid/disodium hydrogen phosphate buffer.) Related to Figure 2.

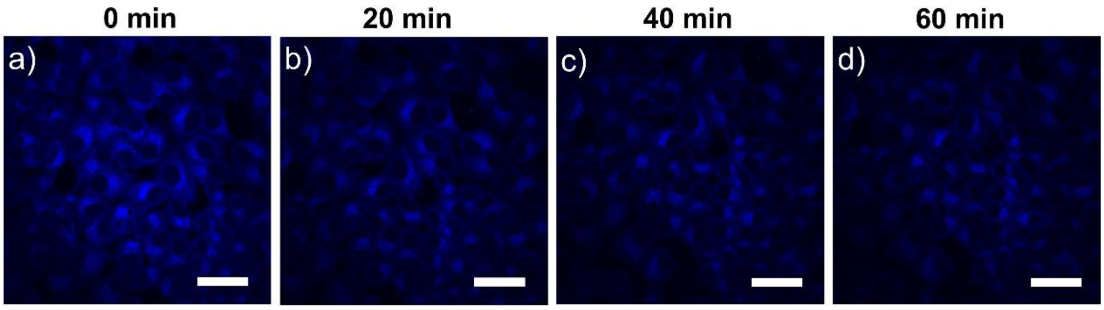

Figure S12. Confocal fluorescence images of A549 cells incubated with CA/oPD-CPDs without treated with dexamethasone. Scale bar: 40 μm. Related to Figure 3.

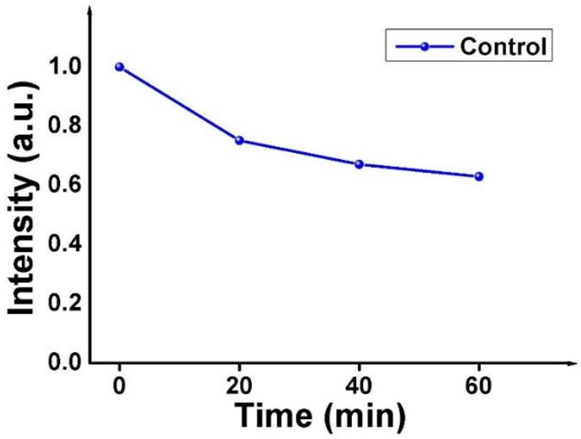

Figure S13. PL intensity of A549 cells incubated with CA/oPD-CPDs without treated with dexamethasone. Related to Figure 3.

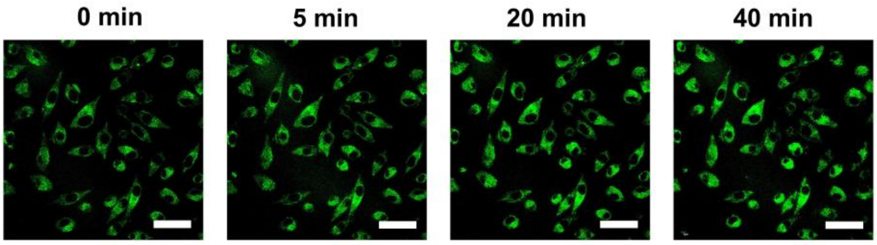

Figure S14. Confocal fluorescence images of living L929 cells incubated with CA/oPD-CPDs under 488 nm laser exposure. Scale bar: 40 μm. Related to Figure 4.

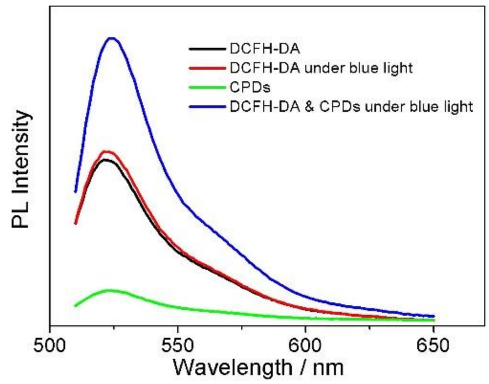

Figure S15. PL spectra of DCFH-DA, DCFH-DA under blue light, CPDs, DCFH-DA with CPDs under blue light. Related to Figure 4.

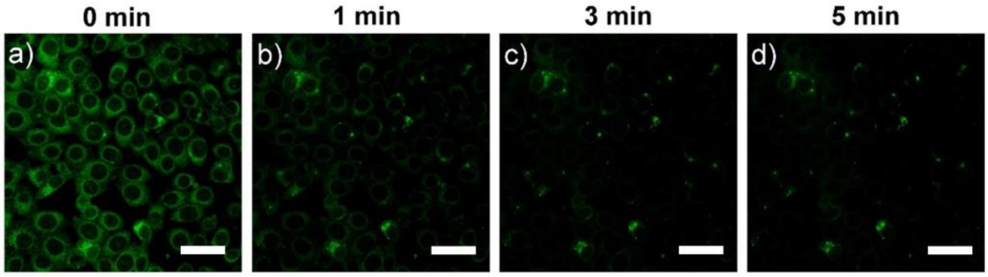

Figure S16. Confocal fluorescence images of fixed A549 cells incubated with CA/oPD-CPDs under 488 nm laser exposure. Scale bar: 40 μm. Related to Figure 4.

Transparent Methods

1. Materials.

Citric acid (99.5%) and o-phenylenediamine (98%) were purchased from Aladdin. Lithium hydroxide monohydrate (90%) was purchased from Beijing reagent. Urea (99%) was purchased from Macklin. Lyso-Tracker Red DND99 was purchased from Invitrogen. Dexamethasone was purchased from Maclin. Human non-small cell lung cancer cell lines A549 and mouse-derived fibroblast cells L929 were purchased from the Academy of Sciences Cell Bank. RPMI medium was purchased from HyClone. Fetal bovine serum and penicillin-streptomycin solution were purchased from Gibco. The Cell Counting Kit-8, CCK-8, was purchased from Dojindo. Cellulose (cotton linter pulp) was supplied by Hubei Chemical Fiber Group Ltd. (Xiangfan, China). Cellulose ester ultra-filtration membranes (0.22 μm) were purchased from Tianjin Navigator Lab Instrument Co., Ltd. Dialysis Membrane (MWCO: 3500 D) are biotech CE tubing and purchased from Spectra/Por Dialysis Membrane. 2',7'-dichlorofluorescein (DCFH-DA) was purchased from Biyuntian Company. All chemicals were purchased and used without further purification.

2. Preparation of CA/oPD-CPDs.

The basic procedures to synthesize CA/oPD-CPDs include two sequential steps in terms of the decomposition and pyrolysis of the mixture of citric acid and o-phenylenediamine. 0.192 g citric acid and 0.108 g o-phenylenediamine were dissolved in the mixture of 10 mL deionized water and 100 μL concentrated hydrochloric acid. The acquired solution was transferred into a poly (tetrafluoroethylene) (Teflon)-lined autoclave (25 mL) and heated at 200 ℃ for 8 h. Then the reactor was cooled to room temperature naturally. The obtained solution was filtrated with ultra-filtration membrane (0.22 μm) to remove larger particles. After filtration, the solution was neutralized with sodium hydroxide, and then centrifuged at 8,000 rpm. The supernatant and the sediment were separately collected. The sediment was transferred to a dialysis bag (3500 D) to remove the remained small molecules for two days in deionized water. The amount of deionized water was 800 mL and the water was changed three times a day. Finally, CA/oPD-CPDs were collected for characterizations and further use. The concentration of CA/oPD-CPDs suspension was determined by the weight of the leftover in 60 ℃ oven per milliliter. The supernatant was purified through column chromatography on silica gel with water as the mobile phase. Batch 2 was the main product and marked as Fluorescent Molecule (FM). Fluorescent Molecule powder was obtained through the freeze-drying process.

3. Cellular toxicity test.

Human lung carcinoma (A549) cells at an initial density of 5×10^3 cells per well were inoculated into the sterile 96-wells plate at 37 ℃ in a humidified 5% CO_2 atmosphere for 24 hours, respectively. Then the culture medium was replaced by a fresh medium with different concentrations of CA/oPD-CPDs (0, 10, 20, 25, 30, 40, 50 μg/mL) and the cells were further incubated for 24 hours. Finally, the samples were washed with PBS, and 100 μL fresh medium with 10 μL CCK-8 was added to each cell well, following by 2 h incubation. A CCK-8 assay was used to analyze cell viability. The optical density was measured at 450 nm. Every condition was repeated for 5 times to gain accurate cytotoxicity data. For the

cytotoxicity of CA/oPD-CPDs with blue light, concentrations of 25 and 50 μg/mL were chosen as typical conditions. To amplify the influence of light exposure, 15 W high powder were applied as long as 2 hours. Without light exposure, the viabilities of cells were 91% (STDEV: 10%) and 79% (STDEV: 16%) when the concentrations were 25 and 50 μg/mL. With blue light exposure, most of A549 cells died. Specifically, the viabilities of cells were 21% (STDEV: 0.18 %) and 17% (STDEV: 0.09%) when the concentrations were 25 and 50 μg/mL.

4. Lysosomes imaging.

Human lung carcinoma (A549) cells were selected for lysosomes bioimaging. A549 cells at an initial density of 2×10^5 cells per well were cultured on a confocal dish for 24 h. Then the cells were incubated with CA/oPD-CPDs with a concentration of 25 μg/mL in a culture medium at 37 ℃ and a humidified 5% CO_2 atmosphere for 24 h. After that, the cells were washed with PBS and further incubated with 100 nM Lyso-Tracker Red DND99 to co-stain the lysosomes in cells for 30 min. After washing and fixing with 2.5% glutaraldehyde for 15 min, the cells were monitored by confocal microscope.

5. Bioimaging of CA/oPD-CPDs during apoptosis.

A549 cells at an initial density of 2×10^5 cells were cultured on a confocal dish overnight. Then the cells were incubated with CA/oPD-CPDs with a concentration of 25 μg/mL for 24 h. After that, 2 μM dexamethasone was added to the culture medium to induce apoptosis and further intracellular pH vibration.

6. Preparation of the composites of CA/oPD-CPDs &cellulose.

As reported in the literature (Wang et al., 2014), 2.4 g LiOH and 4.5 g urea were dissolved in 21.75 mL deionized water. 1.35 g cellulose and 0.2 mg CA/oPD-CPDs were dispersed in the prepared aqueous solution. The whole solution was kept in liquid nitrogen with vigorous stirring until the solution was clear and then centrifuged at 8,000 rpm to degas. The mixture was poured into the designed moulds, and heated at 60 °C for 5 hours. The obtained hydrogel was then soaked in deionized water to remove the excess urea and LiOH.

7. Detection of reactive oxidative species.

The ROS detection was evaluated by the DCFH-DA fluorescent probe. Specifically, 12.5 μL of DCFH-DA was added in 0.5 mL of DMSO and then mixed with 2 mL 0.01 mol L^{-1} NaOH under dark condition. After thirty minutes, the reaction was terminated with 10 mL phosphate buffer (25 mM, pH = 7.2) and DCFH-DA solution was achieved. The four groups were set: control (DCFH-DA solution 1 mL with 1 mL DI water), control with blue light exposure, CA/oPD-CPDs aqueous solution (2 mL DI water with 100 μL 2 mg mL^{-1} CA/oPD-CPDs), and DCFH-DA with CA/oPD-CPDs under blue light exposure (DCFH-DA solution 1 mL with 1 mL DI water and 100 μL 2 mg mL^{-1} CA/oPD-CPDs). Blue light exposure was set as 4 W for 5 min.

8. Characterization.

TEM was conducted using a Hitachi H-800 electron microscope at an acceleration voltage of

200 kV with a CCD cinema. Fluorescence spectroscopy was performed with a Shimadzu RF-5301 PC spectrophotometer. The laser confocal microscopy images were obtained with laser scanning confocal microscope OLYMPUS BX81 (FluoView FV1000). UV-vis absorption spectra were obtained using a Shimadzu 3100 UV-vis spectrophotometer. IR spectra were taken on a Nicolet AVATAR 360 FT-IR spectrophotometer. Absolute QY was measured on a fluorescence lifetime and steady-state spectrometer (Edinburgh Instrument, FLS 920, with an integrating sphere). Raman spectroscopy was done by a LabRAM ARAMIS™ Smart Raman spectrometer (Thermofisher) with a He Ne laser at an excitation wavelength of 785 nm. X-ray Photoelectron Spectroscopy (XPS) was investigated by using ESCALAB 250 spectrometer with a mono X-Ray source Al Kα excitation (1486.6 eV). Binding energy calibration was based on C1s at 284.6 eV.

SUPPLEMENTAL REFERENCE

Wang, Z., Fan, X., He, M., Chen, Z., Wang, Y., Ye, Q., Zhang, H., and Zhang, L. (2014). Construction of cellulose-phosphor hybrid hydrogels and their application for bioimaging. J. Mater. Chem. B *2*, 7559-7566.
